# Evaluation of comprehensive improvement for mild and moderate soil salinization in arid zone

**DOI:** 10.1371/journal.pone.0224790

**Published:** 2019-11-19

**Authors:** Haichang Yang, Yun Chen, Fenghua Zhang

**Affiliations:** 1 Agricultural college, Shihezi University, Shihezi City, Xinjiang, P.R.China; 2 Land and Water, Commonwealth Scientific and Industrial Research Organization, Canberra, ACT, Australia; University of Vigo, SPAIN

## Abstract

Sustainable development of agricultural lands in arid environments is limited by soil salinization. Comprehensive measures were conducted to completely improve soil salinization in this study. For the purpose of assessing the effect of comprehensive improvement in salinized farmland in arid zone, soil salinity at a range of soil depths, EC of subsurface pipe drainage and crop yield during crop growth period in Xinjiang, China were investigated. The results show that soil salinity decreased significantly on mildly (1–3 dS m^-1^) and moderately (3–6 dS m^-1^) salinized farmlands. The improvement in moderately salinized soil was better than that in mildly salinized soil. The average desalinization rate of mildly and moderately salinized farmland was 15% and -15.8%, respectively. The more irrigation times were, the better desalinization effect became. The EC of drainage water varied in the range of 7.53–11.16 dS m^-1^ and was greater than the EC of irrigation water, which showed that subsurface pipe drainage can remove soil salinity from salinized farmlands. The crop yield using comprehensive improvement increased significantly compared with the control check. The outcome of this study suggests that comprehensive measures on salinized farmland are conductive to the decrease of soil salinity and the increase of crop yield.

## Introduction

Salinized soil in Xinjiang, China occupy approximately 33% of the nationwide salinized land area, which has a strong impact on plant establishment, land revegetation and performance of land productivity in arid zone[1–2]. The improvement of soil salinization is necessary to create favorable conditions for land revegetation. Reclamation of salt affected land will be able to raise a locally ecological environment and a healthy stimulate economy development[3].

Numerous investigations have been carried out to solve this soil salinity problem. The approaches involved chemistry measures[4], biological measures[5] and engineering measures[6] to control and monitor soil salt and water content changes. It is essential to study physical and chemical properties of soil as well as crop production with improving soil salinization. In the late 19^th^ and early 20^th^ century, scholars from USA and Russia, such as Hilgard and AHTllnoB. KapaTaeB, built a theoretical equation of soda solonetzic soil, which was improved by gypsum[7] who initiated the precedent of improving soil salinization. Chaganti *et al*.[8] applied chemical improvement to reduce the soil pH and salinity significantly. Yuan *et al*.[9] planted *Achnatherum caragana (Trin*. *et Rupr*.*) Nevski* in salinized soil to increase soil nutrient and microorganism content. Subsurface pipe drainage, furrow irrigation and ditch drainage were considered beneficial to soil salinization improvement[10–11]. Furthermore, other measures such as electromagnetic improvement, soil exchange, decreasing alkali by laying sand, straw mulching, tillage and fertilization achieved the effect of decreasing soil salinity and improving soil quality[12–16]. Nevertheless, the improvement of soil salinization is not only a long-term and complex process, but also connected with many subjects, such as hydrology, meteorology and cultivation[17–18]. There exist a large number of facts affecting soil desalinization process. Therefore, we should take comprehensive measures to improve soil salinization and prevent secondary soil salinization.

Several studies investigated the effect of the single improving treatment on soil salinity variation in the reclamation of abandoned[19–21]. However, single improving treatment is generally regarded incomplete, and knowledge on comprehensive improving treatment at different degrees of soil salinity has not been well understood. Here, we present a field-scale case study which evaluates the impact of comprehensive measures in mild and moderate soil salinization on soil salinity, pipe drainage and crop yield in arid zone of Xinjiang. It contributes to better understanding of the nature and roles of salt and water movement under the condition of comprehensive improvement. The goals of the study are (1) to integrate chemical and biological technologies with engineering technology to comprehensively improve saline-alkali soil, and (2) to assess the impact of soil salinization on the yield and changes in salt content.

## Material and methods

### Study area

**Fig 1:**
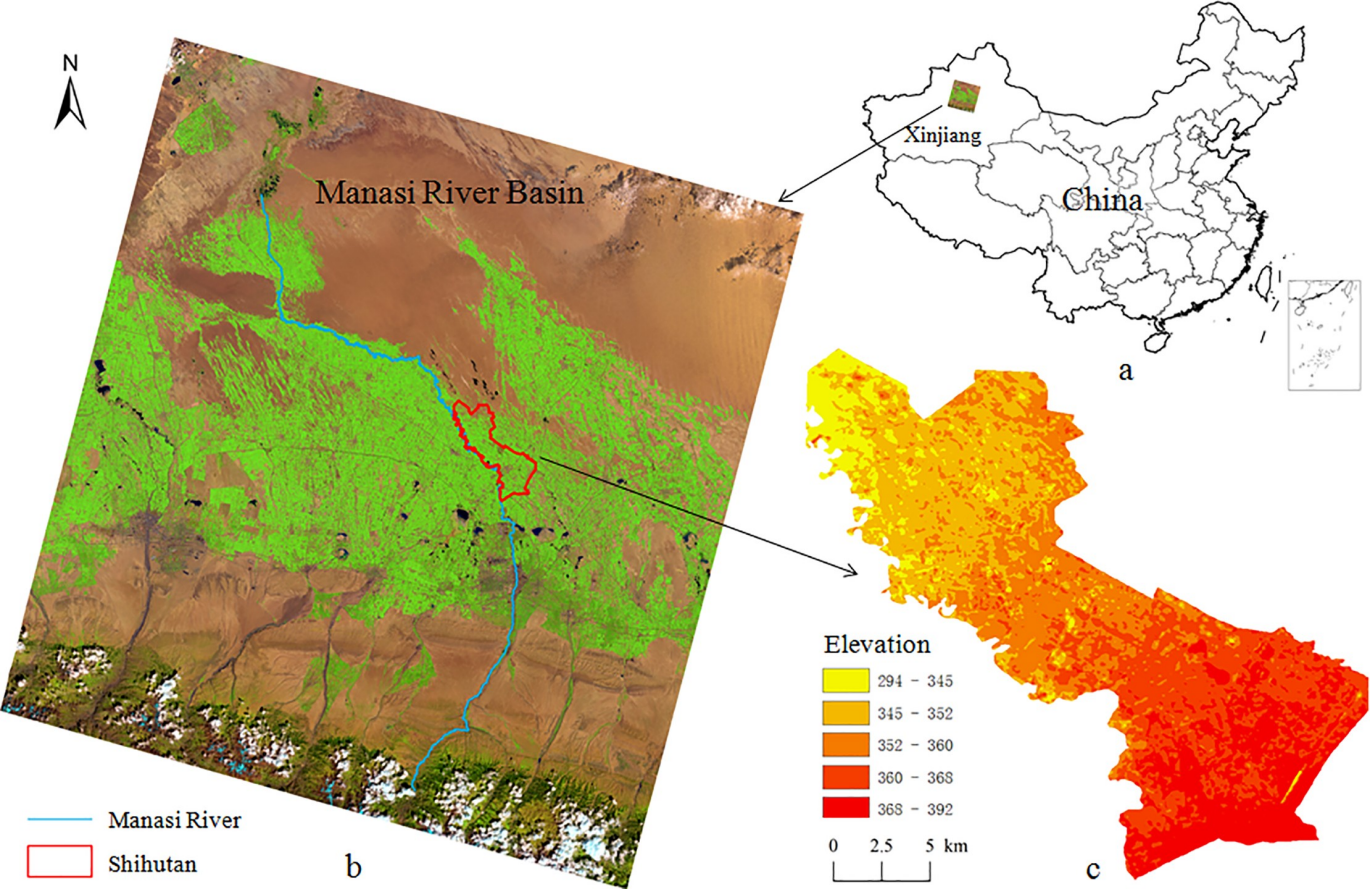
Sketch map of the Manasi River Basin and study region. Note: These images are colour composites of landsat-8 (2014) bands 6, 5 and 2 (RGB) using ARCGIS 10.3 software.

**Fig 2:**
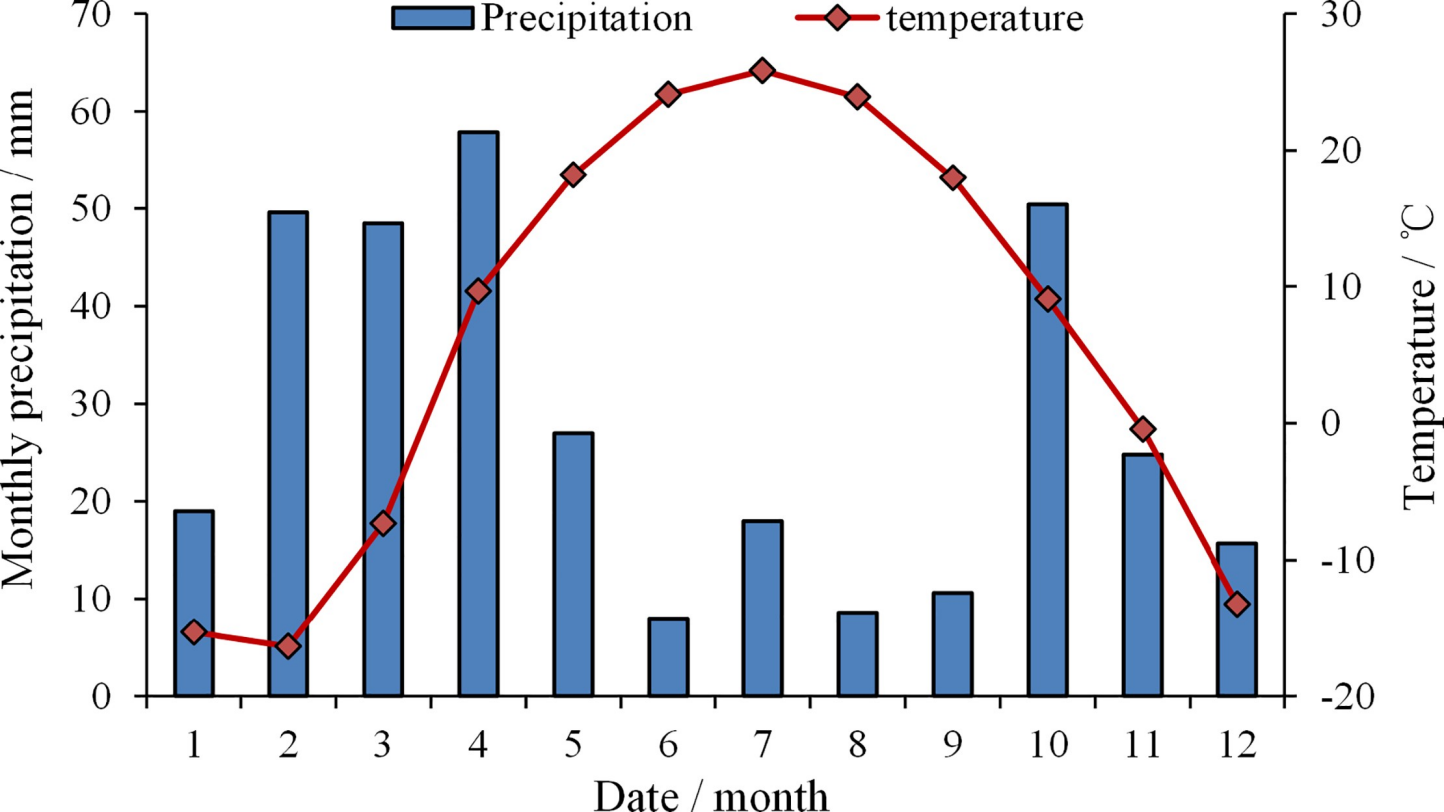
Monthly precipitation and ET in study area.

The Manasi River Basin (44°33′ N, 85°37′ E) is located at the center of Eurasia, along the southern edge of the Junggar Basin of China. The experiment site is a 75 ha area in the Shihutan Township, which lies on the edge of an alluvial fan in the middle of the Manasi River Basin (Fig 1). The depth of groundwater is 1.5–5 m, groundwater mineralization is 5–7 g L^-1^, the average annual temperature is 6.8°C [22]. The average annual precipitation and evaporation are 153 mm and 1255.82 mm from 1956 to 2010 respectively (Fig 2). The mean soil organic matter is 11.4 g kg^-1^ and the mean soil pH is 8.28. Soil type and physical parameter of test area are presented in Table 1. Intense evaporation accelerates the accumulation of salt on the soil surface. Irrational irrigation also exacerbates salinization. A large area of salinized land has been cultivated in Manasi River Basin since 1997, but more than a quarter of farmland was abandoned because of salinization since then.

**Table 1:** Soil type and physical parameter of test area.

Soil profile (cm)	Soil type	Moisture content (%)	Bulk density (g•cm^-3^)	Porosity (%)	Soil pH
0–25	Medium loam	15.33	1.205	54.53	8.30
25–50	Medium loam	16.91	1.559	41.19	8.45
50–70	Heavy loam	21.05	1.601	39.59	8.44
70–90	Heavy loam	20.26	1.582	40.31	8.44
90–110	Sandy loam	22.82	1.716	35.27	8.00
110–130	Medium loam	26.19	1.536	42.04	8.06

### Experimental design

A completely randomized experiment was designed in this study. The experimental cotton field was divided into (i) mild soil salinization as control check (CK), (ii) mild soil salinization with comprehensive improvement, (iii) moderate soil salinization as CK, and (iv) moderate soil salinization with comprehensive improvement. The soil at the site was classified as grey desert soil according to the local classification [23] and Yermosol by the FAO–UNESCO system [24]. The salt content of mild soil salinization is 1–3 g kg^-1^, and the salt content of moderate soil salinization is 3–5 g kg^-1^. Each soil treatment is 25 m^2^ (5m×5m) with 6 replications. The comprehensive improvement measures consisted of biological, chemical and engineering technologies.

#### Biological measure

Cottonseed meal was used as a biological ameliorant on the surface of mild and moderate soil salinization. The application time was one to two weeks earlier than sowing in spring. Application amounts of cottonseed meal are as follows: 1500 kg hm^-2^ in mild soil salinization and 2250 kg hm^-2^ in moderate soil salinization. The soil with cottonseed meal was ploughed 20–40 cm for planting cotton.

#### Chemical measure

Humic acid was used as a chemical ameliorant on the mild and moderate soil salinization. It has a good physiological activity, absorption, complexation and switching functions. The humic acid was applied with irrigation water in seeding emergence and second irrigation. Application amount of humic acid is 75–150 kg hm^-2^.

#### Engineering measure

Shallow subsurface pipe drainage technology was used for decreasing groundwater level and preventing soil salt from moving to the topsoil. Polyvinyl chloride (PVC) corrugated pipe was selected as subsurface pipe material and surrounded by filter. The pipe diameter is 11 cm. The pipes were buried 2 m, and interval spacing of pipes is 90 cm. The procedure is to lay the pipes with filter at the bottom, to put gravel stones above the pipes and then to backfill the upper soil. Drainage well was fixed in the terminal of pipes. The water in drainage well was pumped into the shelterbelt when the suction pump could absorb the water.

Crop in test area was cotton, and the cotton variety was Xinluzao No. 48. Drip irrigation under plastic film was adopted. Film width was 2.1 m with two drip irrigation belts and six rows cotton in each. Wide and narrow rows were adopted in cotton planting, and the widths were 50 cm and 30 cm, respectively. Drip irrigation belts were laid in the middle of wide row. Planting spacing was 15 cm. Parameters of drip irrigation belts were as follows: diameter 16 mm, thickness 0.2 mm, mass per meter 12 kg, dripper diameter 6 mm, dripper spacing 30 cm, dripper flow 2 L h^-1^. Irrigation water came from well, and its mineralization was 0.65 g L^-1^ (freshwater mineralization <1 g L^-1^) and its pH was 7.51. Irrigation times were 11 and total amount of irrigation water was 4500 m^3^ ha^-1^ during the whole cotton growth period, and irrigation average spacing was 9 days.

### Sampling and testing

#### Soil sample collection and testing method

Soil sampling were from each soil treatment was conducted in 2011. The samples were taken in an "S-shaped" pattern (0–40 cm) at six points within each treatment. Sampling point was in the middle of the film. The five samples were mixed and then three 1-kg subsamples which were considered as replications wereremoved for analysis using the “quartering” method. The sampling depths were 0–20 cm, 20–40 cm, 40–60 cm and 60–80 cm. The sample time was seeding stage, budding stage, flowering stage and boll stage.

Moisture content, bulk density, porosity, pH and electrical conductivity (EC) of the soil samples were tested. Soil electrical conductivity and pH were determined in a 1:5 and 1:2.5 soil-water suspension. Soil moisture content was determined using the drying method. Soil bulk density was determined using the cutting ring method. Soil desalinization rate was calculated by using Eq (1):

$$D_{r}=\frac{SS_{T}-SS_{CK}}{SS_{CK}}\times 100\%$$ (1)

where *D*_*r*_ is desalinization rate of soil, *SS*_*T*_ is soil salinity in treatment, *SS*_*CK*_ is soil salinity in CK.

#### Water sample collection and testing method

A water sample in the drainage well was collected by using water collection device. The collected sample was taken to laboratory to test water electrical conductivity, infiltration time and quantity.

#### Cotton yield testing

Cotton yield (kg ha^-1^) was tested from each soil treatment before harvesting. The yield was tested in a diagonal pattern at three points within each soil treatment. These three points were considered to be replications. Testing index included strains (strains ha^-1^), bolls (bolls strain^-1^) and boll weight (g boll^-1^). The cotton yield would be calculated by using Eq (2).

$$Cotton\;Yield=\frac{Strains\times Bolls\times Boll\;Weight}{1000}$$ (2)

### Statistical analysis

Statistical analysis was undertaken using the statistical software SPSS version 20.0 (IBM, Armonk, NY). Data were reported as the mean ± standard error. Least significance difference (l.s.d.) was adopted for a variance analysis[25]. The letter-marking method was used in the comparison of different treatments. Significant differences in EC_s_ among different soil depths were indicated using different lowercase letters (*P* = 0.05). Significant differences in cotton yield between the treatments of subsurface pipe drainage and CK were indicated using different capital letters (*P* = 0.01).

## Results

### Improvement of mild salinization

**Fig 3:**
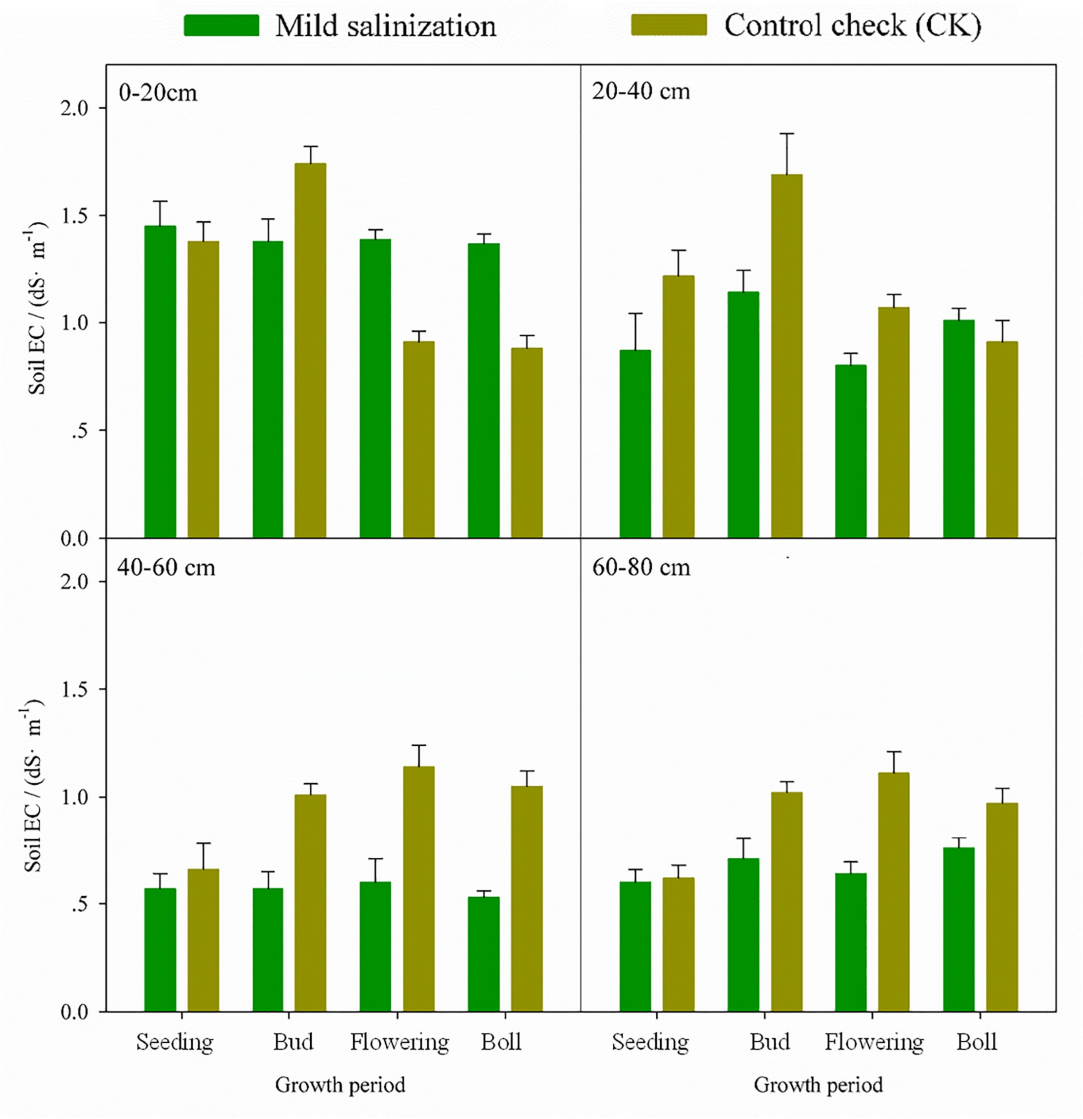
Soil salinity of mild saline area in underground pip. Note: Capital letters mean significant differences in the same layers at 0.01 levels.

Under the condition of comprehensive improving in mild soil salinization soil salinity decreased significantly (Fig 3). Soil salt in 0–20 cm and 40–60 cm layers showed a trend of lower volatility along with the growth of cotton plants. However, soil salt in 20–40 cm and 60–80 cm had an opposite trend, and the range in 20–40 cm was much higher than other layers. Soil salt transformation rules varied in the different layers, because evapotranspiration and irrigation volume changed in different period. The CK was not treated by the comprehensive treatments in mild soil salinization to remove the effect of other environmental factors on soil salt transportation.

Soil average desalination rate of mild saline area was -15% (Table 2). The rate reduced after the first increased along with the growth of cotton plants. It’s increase in 0–20 cm layer along with the growth of cotton plants. It decreased in other layers with the largest rate occurring in 20–40 cm depth. These prove that the comprehensive measures can significantly decrease soil salinity. However some part of soil salinity gathered in the soil surface. One reason could be due to the large evaporation in Xinjiang oasis. Soil salt moved to the soil surface with water. Besides, chemical fertilizer along with the drip irrigation into farmland would increase soil ions content. Soil desalination rate in vegetative growth period was higher than that in reproductive growth period after a comprehensive improvement, particularly in budding stage. A lower salinization environment was provided to increase cotton biomass in budding and flowering stage. It would narrow the cotton-yield gap between mild soil salinization and control check.

**Table 2:** Soil desalination rate of mild saline area.

Sampling time	0–20 cm(L1)	20–40 cm(L2)	40–60 cm(L3)	60–80 cm(L4)	D_r_
seeding stage	4.7	-28.7	-14.4	-4.0	-10.6
budding stage	-21.0	-32.5	-44.1	-30.4	-32.0
flowering stage	52.7	-25.7	-47.8	-42.3	-15.8
boll stage	55.1	10.4	-49.5	-22.2	-1.5
average	22.9	-19.1	-38.9	-24.7	-15.0

units: %

### Improvement of moderate salinization

**Fig 4:**
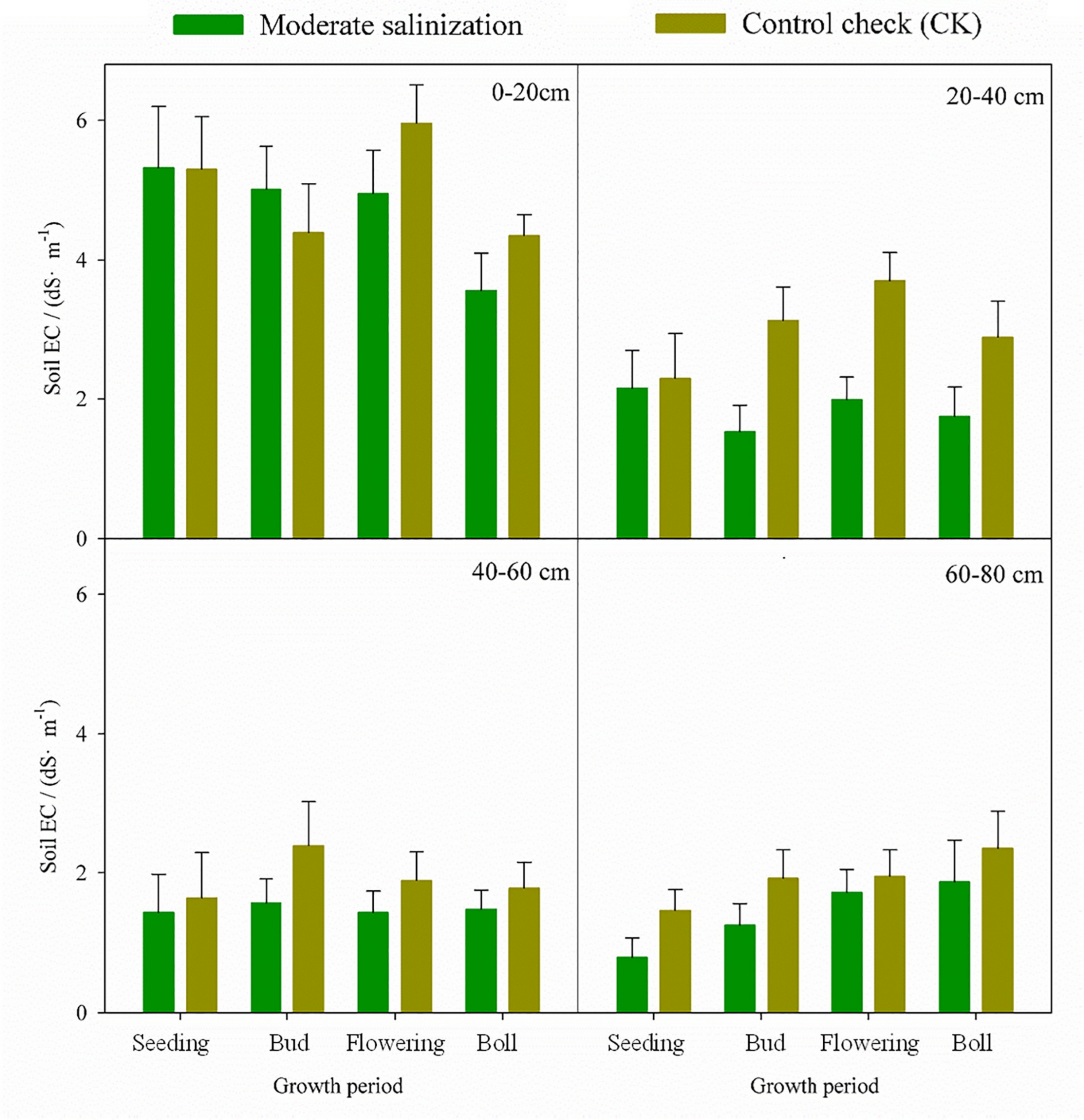
Soil salinity of moderately saline area in underground pipe. Note: Capital letters mean significant differences in the same layers at 0.01 levels.

Under the condition of comprehensive improving in moderate soil salinization soil salinity decreased significantly (Fig 4). Soil salt in 0–20 cm and 20–40 cm layers showed a trend of lower volatility along with the growth of cotton plants. Soil salt in 40–60 cm changed slowly. However, soil salt in 60–80 cm had an opposite trend. Comprehensive treatments could decrease soil salt in the root zone, and cause a large number of salt to accumulate below the root zone. Comprehensive treatments could eliminate the salt effect on cotton growth. The depth of soil salt accumulation depended on the irrigation water volume. If the irrigation water volume was large enough to make sure that the salt could move out from the farmland through the pipe drainage system. If the irrigation water volume was not enough, soil water flux would be decreased in the deep soil layer, salt would accumulate there and then move up to the surface later. Unreasonable irrigation and tillage model could result in the secondly salinization.

Soil average desalination rate of moderate saline area was -15.8% which is slightly that of higher than mild saline area (Table 3). Soil desalination rate in all layers decreased with the largest average rate of -35.6% observed in 20–40 cm. Soil desalination rate increased along with the growth of cotton plants. The comprehensive measures could significantly decrease soil salinity. The effect of soil desalination became better and better with the increase of irrigation times in the course of soil desalinization using comprehensive measures. In addition, comprehensive measures could prevent salt from returning to the surface and inhibit soil secondary salinization.

**Table 3:** Soil desalination rate of moderate saline area.

Sampling time	0–20 cm(L1)	20–40 cm(L2)	40–60 cm(L3)	60–80 cm(L4)	D_r_
seeding stage	0.4	-6.1	-12.8	-8.7	-6.8
budding stage	14.1	-51.0	-34.3	2.5	-17.2
flowering stage	-15.5	-46.2	-10.1	-2.0	-18.4
boll stage	-18.4	-39.2	-16.9	-9.0	-20.9
average	-4.9	-35.6	-18.5	-4.3	-15.8

units: %

### Subsurface pipe drainage

**Fig 5:**
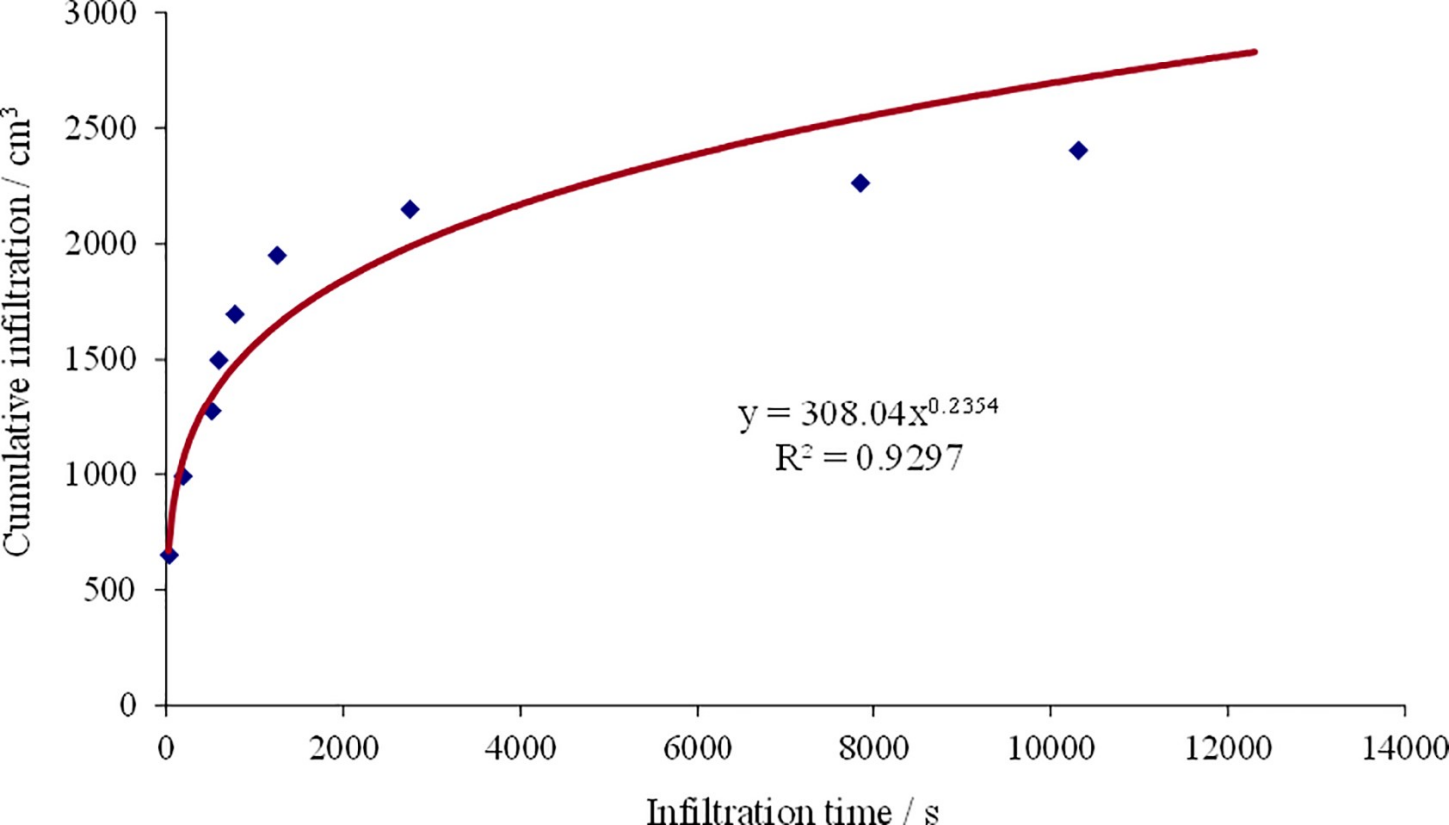
Change regularity of soil infiltration rate after irrigation.

After irrigation under the condition of putting the comprehensive measures into practice, soil infiltration rate was significantly correlated with infiltration time (Fig 5). According to the Kostiakov formula which describes the soil water infiltration characteristics, the equation of cumulative infiltration was simulated as Eq (3):

$$I=308.04t^{0.2354}$$ (3)

In this formula, t means soil cumulative infiltration time.

Soil infiltration rate in farmland was affected by irrigation mode, cultivation management measures and precipitation. Drip irrigation under plastic film was implemented in large scale areas in Xinjiang where is China’s most arid area and had the widest distribution of saline soil, so soil infiltration rule was different from others.

**Fig 6:**
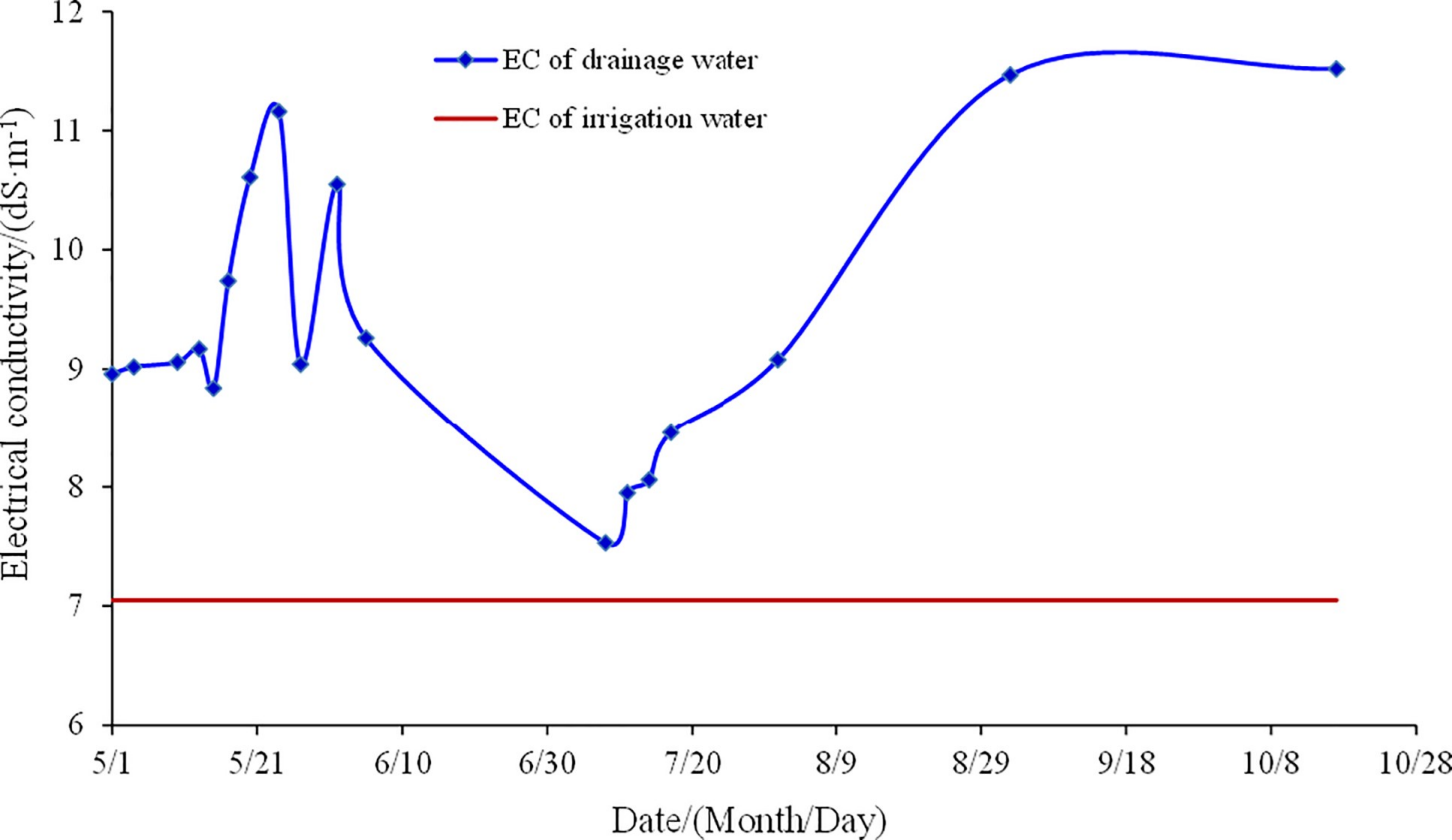
Salt dynamics of drainage water from underground pipe.

In order to analyze the effect of comprehensive measure, the pipe drainage was collected to test its electrical conductivity (EC) (Fig 6). The EC of the pipe drainage presented a trend of “increased-decreased-increased”, range of pipe drainage EC was 7.53–11.16 dS m^-1^ during the whole growth period. Compared with the initial value, pipe drainage EC changed from -17.9% to 27.6%. Since the farmland irrigation water came from confined water and its salinity was low, soil salinity resulted from irrigation water was very little. According to the pipe drainage EC change after farmland was irrigated in May 20th, June 12th and July 8th, irrigation water could leach soil salt. Soil salt in mildly salinized farmland decreased in the whole soil layers, except that the surface layer showed an increasing trend. Soil salinity in moderately salinized farmland presented the effect of desalination in all the layers. Therefore, soil desalinization was closely related to the subsurface pipe drainage. Drip irrigation water under plastic film could leach soil salt to the deep soil and discharge soil salt from the farmland through underground pipes.

### Cotton yield and efficiency analysis

**Fig 7:**
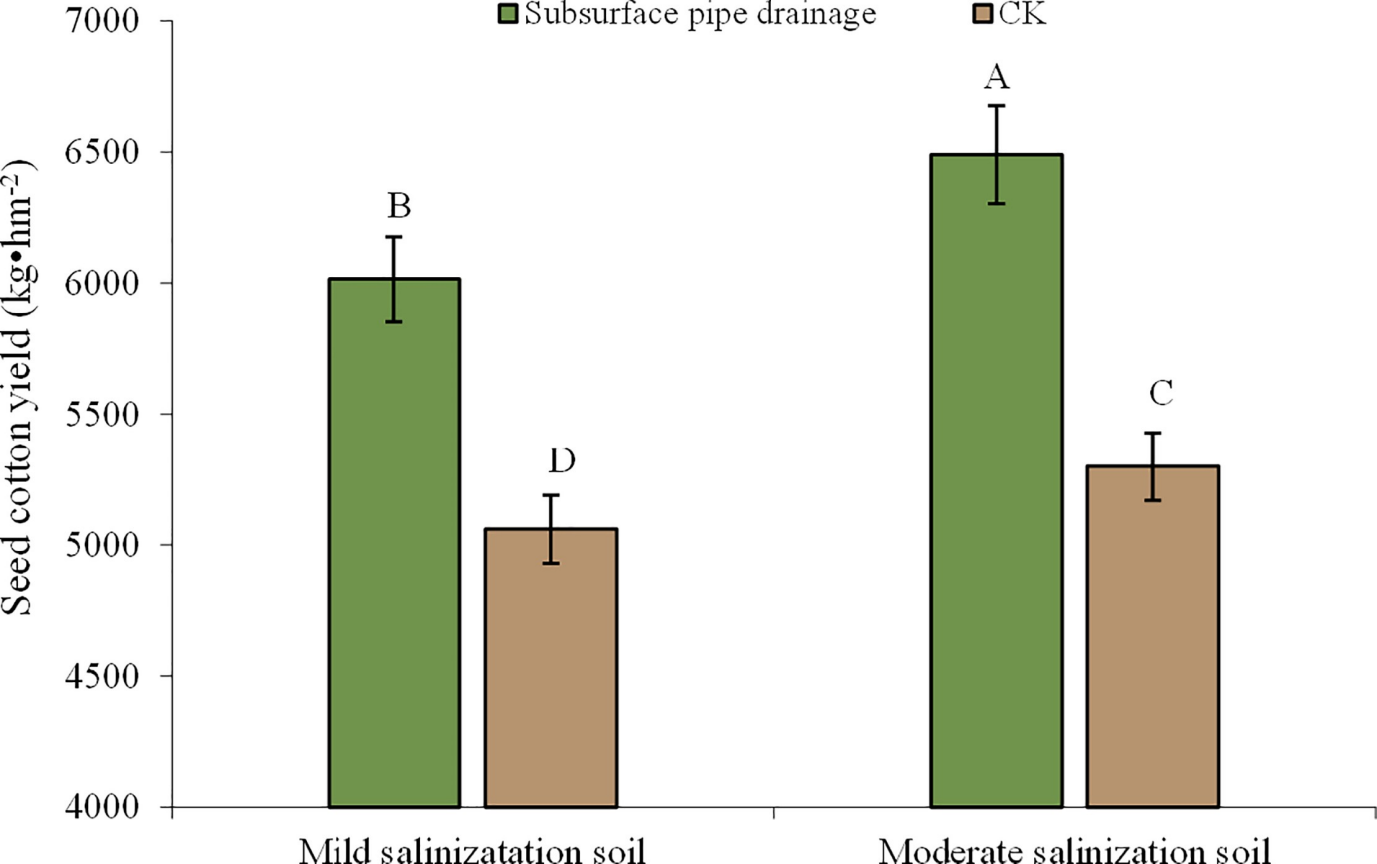
Changes of cotton yield under different salinization field. Note: Different capital letters mean significant differences between treatments and CK at 0.01 level.

Variance analysis showed that the cotton yield in mild or moderate soil salinization was significantly different compared to the CK (P<0.01) (Fig 7). Comprehensive measures could improve cotton yield significantly. The cotton yield in mildly salinized farmland increased by 25.3%, and the cotton yield in moderately salinized farmland increased by 55%. On the surface, the comprehensive measures could also improve crop growth: salt speckles decreased significantly, rate of emergence changed from 20% to 70%, and dead seeding rate reduced significantly.

## Discussions

### Comprehensive measures and desalination effects

Previous researches and findings indicated that using a single technology for improvement of saline soil had some shortcomings: large amount of labor employment, high salary cost and soil secondary salinization[26–27]. Comprehensive measures could completely discharge soil salt of farmland, largely increase soil microbial quantity, and remarkably improve nutrient content and soil fertility, so as to ultimately achieve the goal of increasing crop yield[28]. Comprehensive measures also changed soil ecology environment fundamentally and were more sustainable than single improvement method. However, improving model of comprehensive measure on soil salinization should have a further study and discussion[29]. Therefore, this study focuses on improving abandoned salinized farmland using comprehensive measures. Results proved that comprehensive measures could decrease soil salinity and increase crop yield, which was consistent with previous studies[30–31]. However, the study was limited by the expensive labor-cost. Several issues, such as impact of crop yield increasing on the variation of soil physicochemical and biological characteristics, need to be future addressed. These variations included changes of soil structure, soil organic matter, soil moisture and temperature, and soil microorganism.

### Salt accumulation on soil surface

Comprehensive measure could effectively reduce soil salinity in mildly salinized farmland. However, salt accumulation on soil surface came up. A possible explanation was that evapotranspiration in Xinjiang province was larger than other place. As irrigation times increasing, soil salt was leached to deep soil layer. If soil salt could not be discharge from farmland immediately, soil salt would move to topsoil with the help of crop evaporation and soil surface evaporation, then soil secondary salinization occur. Up to the present, comprehensive measures were considered as the best method on soil secondary salinization improvement in China and aboard. In this study, increasing irrigation amount was the most effective approach to prevent soil secondary salinization.

### Improving effect comparison between mildly and moderately salinized soil

Soil salinity of the mildly and moderately salinized cotton field showed significant variation after a comprehensive measure. Soil desalinization rates in mildly and moderately salinized farmland were 15% and 15.8% compared with CK treatment. Salinity of the moderately salinized soil decreased during all soil layers. The desalinization rate of soil at L2 which was the largest decrease among all layers was -35.6%. Salinity of the mildly salinized soil increased in L1 and decreased in other layers. The decrease of soil salinity at L3 was greater than those at other layers, and the desalinization rate of soil was -38.9%. Improving effect in moderately salinized farmland was better than that in mildly salinized farmland. Crop yield indicated that the yield increasing effect in moderately salinized farmland was greater than that in mildly salinized farmland.

### Economic benefits

Comprehensive measures could effectively reduce 15% soil salinity and achieve the goal of desalinization. The utilization of subsurface pipe drainage saved 5%-8% farmland, and improved the oasis land sources. Furthermore, human and financial resources were also saved because of eliminating dredging of open drain year after year.

The benefit of ecological environment was appreciable in moderately salinized farmland with higher groundwater level after subsurface pipe drainage was conducted. Soil salinity and groundwater level in mildly and moderately salinized plot was reduced significantly. It was more likely that soil salinity moved down with irrigation water into the shallow groundwater, and then saline groundwater was discharged through pipe drainage. This technology fundamentally solved salt accumulation in farmland and eliminated salt accumulation throughout the year at soil depth of under 60 cm. The average desalinization rate of the mildly and moderately salinized soil was 19.2%-51.0%. Furthermore, upper land of drainage pipe could be used to farm as normal. Drain pipe was laid one time and could be used for many years. These results indicate that comprehensive measures with pipe drainage was effective to decrease soil salinization and increase economic benefit.

## Conclusions

The effect of comprehensive measures on mildly and moderately salinized farmland was examined in an integrated analysis of soil salinity, drainage water and crop yield during crop growth stages under mulched drip irrigation. The study results have proved that the improvement effect in moderately salinized soil is better than that in mildly salinized soil. Soil salinity in the mildly and moderately salinized plot was reduced to the acceptable range for crop growth in 0–40 cm soil layer. The average desalinization rate of mildly and moderately salinized farmland was 15% and -15.8%, respectively. The EC of drainage water varied in the range of 7.53–11.16 dS m^-1^ and was greater than the EC of irrigation water. Comprehensive measures could significantly improve cotton yield. Compared with the CK, the yield in mildly salinized farmland increased by 25.3%, and the yield in moderately salinized increased by 55%.

Finally, the field experiment was limited by the labor-cost. Several issues need to be further investigated to ensure adequate and detailed information on soil salinization improvement. These include impacts of different improvement measures, different irrigation water amount and different crop types. This comprehensive approach has provided an effective technical basis to policy making on desalinization, land-saving, improving fertility and crop yield in arid region.
